# Quantification of IgY to *Erysipelothrix rhusiopathiae* in serum from Swedish laying hens

**DOI:** 10.1186/s12917-021-02813-0

**Published:** 2021-03-06

**Authors:** Eva Wattrang, Helena Eriksson, Ann Albihn, Tina Sørensen Dalgaard

**Affiliations:** 1 Department of Microbiology, National Veterinary Institute, Uppsala, Sweden; 2 Department of Animal Health and Antimicrobial Strategies, National Veterinary Institute, Uppsala, Sweden; 3 Department of Biomedical Science and Veterinary Public Health, Swedish University of Agricultural Sciences, Uppsala, Sweden; 4 Department of Animal Science, Aarhus University, Tjele, Denmark

**Keywords:** Erysipelas, *Erysipelothrix rhusiopathiae*, Laying hen, IgY

## Abstract

**Background:**

Erysipelas, caused by *Erysipelothrix rhusiopathiae* (ER), is an important emerging disease in free-range and organic egg-production. The aim of the present study was to assess if quantification of ER specific IgY titers may aid the understanding of erysipelas in commercial laying hens. The methodology was validated with sequentially collected sera from experimentally ER infected SPF-chickens and subsequently applied on sera from Swedish commercial laying hens collected during and after outbreaks of erysipelas or collected at slaughter from healthy hens housed in furnished cages, barn production or in organic production (with outdoor access).

**Results:**

In experimentally infected SPF-chickens, titers to ER were significantly increased approximately one week after infection while IgY to ER in uninfected age-matched controls remained low. Also chickens infected with low doses of ER, not displaying clinical signs of disease and with low recovery of ER in blood samples showed high titers of IgY to ER. For laying hens during and after erysipelas outbreaks the majority of samples were considered positive for antibodies to ER with a large variation in levels of IgY titers to ER between individuals. For healthy laying hens at slaughter all samples were deemed positive for antibodies to ER. An influence of flock on levels of IgY titers to ER was observed for both healthy hens and hens during erysipelas outbreaks. For healthy laying hens at slaughter no influence of the housing systems included in the study, history of erysipelas outbreaks at the farm or vaccination on levels of IgY titers to ER was noticed.

**Conclusions:**

Taken together, these results show that high numbers of commercial laying hens showed high IgY titers to ER, comparable to those elicited by experimental ER infection, indicating that ER or bacteria that raises antibodies that cross-react with ER are common in this environment.

## Background

Erysipelas is an emerging infectious disease in commercial laying hens housed in cage-free systems particularly those with outdoor access [1–10]. The disease is caused by the bacterium *Erysipelothrix rhusiopathiae* (ER), which is a Gram-positive facultative anaerobic rod. It may infect a very wide range of hosts for example many mammalian species including humans as well as many avian species with or without causing clinical disease (reviewed in [11]). Erysipelas was first described in the late nineteenth century but knowledge on many basic aspects regarding this infection including the epidemiology, route of infection, immunity to infection and prophylaxis, remain very limited (reviewed in [11–13]) particularly regarding chickens. In general, even though ER may survive in the environment for some time, asymptomatic carriers are considered the most important reservoir for the bacterium [12].

In laying hens the disease manifests as acute outbreaks with a rapid progression and a high mortality of up to 60 % [1, 3, 4, 14]. Apart from mortality, affected flocks also often display egg production losses. Clinically the disease in laying hens present with unspecific signs of acute septicaemia such as decreased appetite, depression, weakness, ruffled feathers and drooping wings. *Post-mortem* macroscopic findings are also indicative of septicaemia such as splenomegaly, hepatomegaly, petechial hemorrhages on internal organs and occasional valvular endocarditis. Diagnosis is confirmed by isolation of ER from affected chickens. The source of ER and route of infection in these outbreaks remain largely unknown but erysipelas in laying hens has been associated with floor housing with a higher incidence in flocks with outdoor access [1–10]. In addition, a recent analysis of erysipelas outbreaks in Sweden between 2010 and 2019 showed that 67 % of outbreaks occurred in flocks from 60 weeks of age and upwards (manuscript in preparation). In Sweden, laying hen flocks affected by an erysipelas outbreak are often culled for animal welfare reasons. To prevent new outbreaks on the affected farms subsequent flocks are commonly vaccinated at placement with a single dose of an inactivated vaccine licensed for turkeys [7]. On the whole, this strategy is perceived to be effective although outbreaks in vaccinated flocks have also been reported [7] and analysis of erysipelas outbreaks in Sweden between 2010 and 2019 showed that 30 % of outbreaks occurred in vaccinated flocks (manuscript in preparation). Regarding prevalence of ER in chickens, we have previously found that during erysipelas outbreaks, ER was readily detected in the diseased chickens and their environment [5, 7] while both chicken and environmental samples from two unaffected laying hen farms were negative for ER [7]. On the other hand, one early report described isolation of ER from laryngeal samples from healthy African backyard chickens [15]. In addition, two Japanese studies of healthy chickens at slaughter showed that slaughter bi-products from 83 % of farms tested were positive for *Erysipelothrix* spp [16] and that 30 % of chicken meat samples were positive for *Erysipelothrix* spp [17] by culture methods, respectively, and in both studies the majority of positive isolates were ER. Moreover, three serological surveys of healthy chickens revealed relatively high levels of birds positive for antibodies to ER [6, 18, 19]. A report on laying hens at slaughter in Japan showed 5.5 % positive chickens [18], while a report on laying hens at slaughter in Sweden, showed 100 % positive chickens [6] and a report on chickens of different age and production categories in New Zealand showed overall 40 % positive chickens [19]. Thus, the acute onset and fulminant progression of erysipelas outbreaks in laying hen flocks may suggest that ER was introduced into a previously naïve population while some of the limited bacteriological and serological evidence from healthy birds on the other hand suggests that that ER may be quite common in the chicken environment.

In addition to simply testing for presence or absence of antibodies to an infectious agent quantification of antibody levels may provide additional information such as indications of level of exposure and estimates of time since exposure. Previous surveys of antibodies to ER in samples from commercial chickens using ELISA methodology [6, 19] have used one-dilution systems with positive/negative as the main read out. The aim of the present study was therefore to explore if an ELISA methodology developed for optimal quantification of antibody levels would provide additional information of value when studying erysipelas in commercial laying hens such as insights into ER exposure or vaccination responses. For this purpose we applied an ELISA method recently set up for quantification of ER IgY levels using 2-fold titration of samples and calculating titers using regression analysis in the interval where the relationship between sample dilution and colour reaction was linear [20]. This assay was originally evaluated to samples from an experimental ER infection of SPF-chickens [20]. For further assessment of IgY titers to ER in chickens infected under experimental conditions we included additional samples from a set of experimental ER-infections previously performed [21] in the present analysis. The assay was then applied to cohorts of serum samples from commercial laying hens during and after outbreaks of erysipelas and of serum samples from slaughter of healthy commercial laying hens housed in different systems. Some of these healthy hens originated from farms that previously had experienced outbreaks of erysipelas and some of these hens were vaccinated against erysipelas at placement on the farm.

## Results

### Evaluation of the ER-IgY ELISA with samples from experimentally infected SPF-chickens

**Fig. 1:**
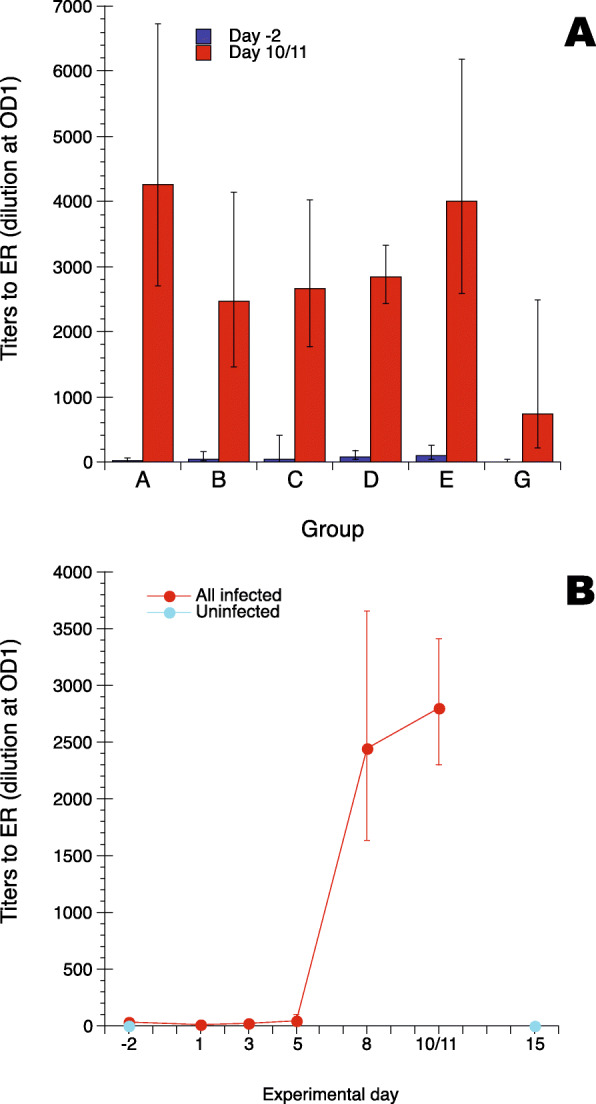
Titers to the “intermediate” ER antigen in serum from uninfected and experimentally ER infected SPF-chickens. **a** Titers to ER in serum collected on day − 2 (blue bars) and day 10/11 (red bars) from six groups of chickens infected with ER on day 0. Groups A, B and G *n* = 13, group C *n* = 11, groups D and E *n* = 18.  **b** Kinetics of titers to ER in serum from chickens infected with ER on day 0 (red circles) and from uninfected chickens, i.e. group F (blue circles). Infected chickens: day − 2 *n* = 86; day1 and 5 *n* = 45; day 3 and 8 *n* = 41; day 10/11 *n* = 80. Uninfected chickens day − 2 and day 15 *n* = 13. All values are group geometric mean values ± 95 % CI where values with non-overlapping CI were interpreted as being statistically different. Due to technical reasons groups D and E were sampled on day 10 instead of day 11, groups F and G were sampled on day − 3 instead of day − 2 and group F was sampled on day 15 instead of day 11. Antibody titers for groups F and G have earlier been presented [20]. For details on chicken groups see Table 1

The ELISA for ER-specific IgY was evaluated using sequentially collected serum samples from six groups of experimentally infected SPF-chickens and one group of uninfected SPF-chickens (Table 1). For these samples an ER antigen derived from the ER strain used for infection was used. This strain was classified as belonging to “intermediate” lineage according to the system described by Forde et al. [22] and termed herein as the “intermediate” ER antigen. All infected chickens except two in group C and one in group E showed significant increases in IgY titers to ER compared to pre-infection titers from day 8 after infection and onward. The three chickens that did not show any ER specific IgY responses were omitted from further analysis of the dataset. Analysis of IgY titers to ER pre-infection, day − 2, and day 10/11 for the different infection groups (Fig. 1a) showed relatively high individual variation in the IgY levels both before and after infection. Before infection varying levels of probably maternally derived antibodies were detected in all groups of chickens. After infection a statistically significant increase in IgY titers to ER was observed for all six groups of infected chickens. Within group comparison of IgY titers to ER in serum collected pre-infection, day − 2, with day 10/11 after infection showed that the mean increase in IgY response varied between approximately 220-fold (group A) to approximately 30-fold (group D). At day 10/11 after infection the mean IgY titers to ER varied between the different groups with group G, infected with the highest infection dose tested of 0.5 × 10^10^ cfu ER/chicken, showing the lowest titres while groups A and E, infected with 0.5 × 10^5^ and  1.6 × 10^6^ cfu ER/chicken, respectively, showing the highest titers. These differences in IgY titers to ER were however not statistically significant between all groups. Analysis of ER-specific IgY responses for all infected chickens irrespective of infection dose (Fig. 1b) showed that all chickens had statistically significant increased titers on day 8 after infection and that by day 10/11 after infection, titers were approximately 1000-fold higher than those of age-matched uninfected control chickens.

**Table 1:** Description of experimental ER infections of chickens. Chickens were infected with the indicated number of bacteria (Dose) by intramuscular injection on experimental day 0 and blood was collected on the indicated experimental days

Trial	Group ID	Dose (cfu/chicken)	n/group	Age at day 0 (days)	Sampling days
1	A	0.5 × 10^5^	13	22	-2, 1_a_, 3_a_, 5_a_, 8_a_, 11
1	B	0.5 × 10^6^	13	22	-2, 1_a_, 3_a_, 5_a_, 8_a_, 11
1	C	0.5 × 10^7^	13	22	-2, 1_a_, 3_a_, 5_a_, 8_a_, 11
2	D	1.6 × 10^8^	18	26	-2, 1_a_, 3_a_, 5_a_, 8_a_, 10
2	E	1.6 × 10^6^	19	26	-2, 1_a_, 3_a_, 5_a_, 8_a_, 10
3	F	Uninfected	13	30	-3, 15
3	G	0.5 × 10^10^	13	30	-3, 1_a_, 3_a_, 5_a_, 8_a_, 11_a_

These infection experiments have previously been described in detail [20, 23]

_a_ - the experimental groups were divided into halves (*n* = 7 respectively 6/group) and individual chickens were only sampled at every other occasion to limit the impact of repeated blood sampling

Thus, the ELISA detected clear differences in ER-binding IgY levels between infected and uninfected SPF-chickens. Infected chickens showed high IgY titers to ER from one week after infection and uninfected SPF-chickens had very low titers throughout the experiment, i.e. to 45 days of age.

### IgY titers to ER in sera from laying hens during and after outbreaks of acute erysipelas

**Fig. 2:**
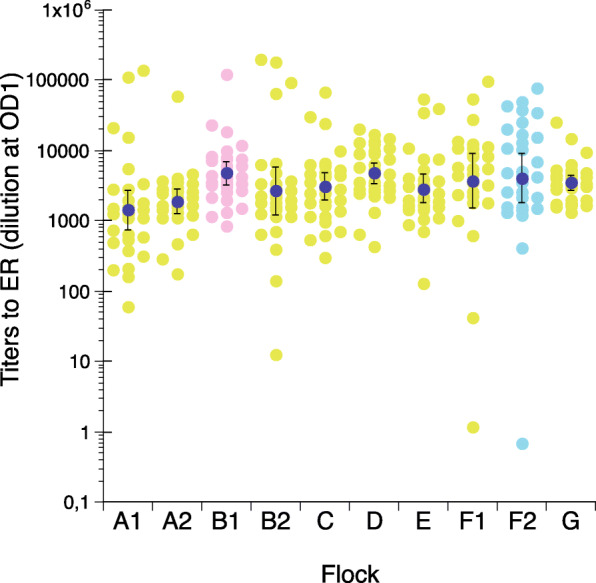
Titers to the “intermediate” ER antigen in serum from laying hens in the indicated flocks during or after acute erysipelas outbreaks. Individual values for hens during acute outbreaks (light green circles), from hens one week after initial sampling (light blue circles) and from hens six months after acute disease (pink circles) and flock geometric mean value ± 95 % CI (dark blue circles). For details on flocks see Table 2

Serum samples were collected from eight laying hen flocks during acute erysipelas outbreaks and one flock (B1) six months after an acute outbreak of erysipelas (Table 2) and tested for IgY titers to ER in the ELISA using the “intermediate” ER antigen (Fig. 2). One of these flocks was also re-sampled one week after the initial sampling (flock sample F2). The antigen was chosen as a representative of recent erysipelas outbreaks among Swedish laying hens since the classification of isolates recovered from the current flocks were unknown at the time of analysis. Overall the majority of samples from these flocks were considered positive in comparison with the results from the experimentally infected SPF-chickens with in total only five samples with a titer of < 100. In most flocks, a large variation in antibody levels between individual samples was observed with a number of individuals both considerably below and above the flock mean titer value. Comparison of flocks sampled during acute outbreaks of erysipelas also showed some between-flock variation in antibody levels where the flock mean titer value for A2 was statistically significantly lower than those for B1 and D. Of the flocks sampled during acute outbreaks of erysipelas, flock F1 had the highest proportion of samples with high titers of > 10,000 (30 %) while flock A2 had the lowest proportion of such samples (3 %). Flock B2 had the highest proportion of samples with low titers of < 1000 (17 %) while flock G had no such samples. The flock on farm F was re-sampled 1 week after the first sampling and the flock mean titer value was not statistically significantly different on the second sampling compared to the first although the proportion of samples with high titers of > 10,000 was higher (40 %) compared to the first sampling. The flock mean value for A1, sampled six months after an acute outbreak of erysipelas, was statistically significantly lower than those for flock B1 and D and had the highest proportion of samples with low titers of < 1000 (37 %).

**Table 2:** Description of layer flocks with outbreaks of acute erysipelas

Farm	Flock sample ID	Housing system	Age at sampling (weeks)	Number of samples	Classification of outbreak ER isolate^a^	Comment
A	A.1	Organic	77	30	Clade 3	Samples collected during an acute outbreak.
A	A.2	Organic	55	29	ns	Samples collected during an acute outbreak in flock subsequent (1 year later) to flock A.1.
B	B.1	Organic	75	31	Clade 3	Samples collected from surviving hens 6 months after an acute outbreak.
B	B.2	Organic	55	29	Clade 3	Samples collected during an acute outbreak in a flock subsequent (1 year later) to flock B.1.
C	C	Organic	72	31	ns	Samples collected during an acute outbreak.
D	D	Free-range	90	30	Clade 2	Samples collected during an acute outbreak.
E	E	Organic	50	30	ns	Samples collected during an acute outbreak.
F	F.1	Organic	66	27	ns	Samples collected during an acute outbreak.
F	F.2	Organic	67	30	nt	Samples collected 1 week after F.1 in the same flock.
G	G	Free-range	78	30	Intermediate	Samples collected during an acute outbreak.

*ns *not sequenced

*nt *not tested

^a^Some of the ER isolates collected from outbreaks were whole genome sequenced within another project and assigned to clades according to whole-genome SNP comparison with isolates from the study by Forde et al. [21]. These data were however not available to us at the time of ER antibody analysis

Thus, IgY to ER was detected in serum in the majority of chickens in laying hen flocks sampled during or after outbreaks of acute erysipelas.

### IgY titers to ER in sera from laying hens at slaughter

**Fig. 3:**
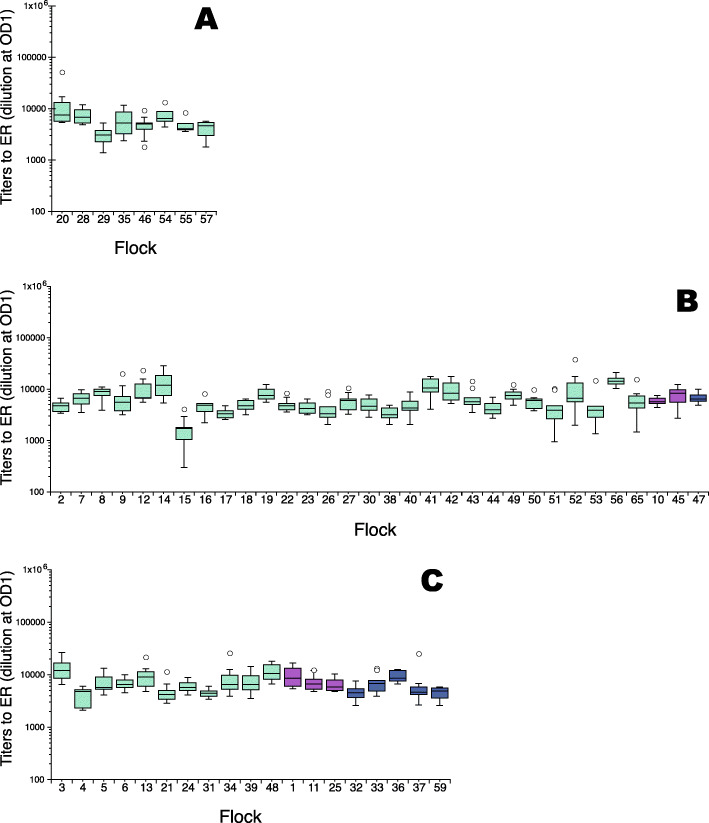
Titers to the clade 2 “vaccine related” ER antigen in serum from laying hens at slaughter. **a** Flocks housed in furnished cages (**b**) Flocks housed in barn production and (**c**) Flocks housed in organic production with outdoor access. Flocks were from farms with no previous outbreak of erysipelas (turquoise boxes) or from farms that had previous outbreaks of erysipelas that either did not vaccinate hens (purple boxes) or that vaccinated hens against erysipelas (dark blue boxes). Data is shown as flock box plots with 10 ≥ n ≤ 8 for each flock. Boxes enclose 50 % of the data with the median value displayed as a horizontal line and the limits of the box represent the upper and lower quartile. Whiskers mark the maximum and minimum values excluding outliers. Open circles represent outliers defined as values greater than the upper quartile, or smaller than the lower quartile, + 1.5x the interquartile distance

Five hundred and sixty one serum samples collected at slaughter of laying hens from 59 flocks housed either in furnished cages, in barn production or in organic production with outdoor access were tested for IgY titers to ER (Fig. 3). The antigen used in the ELISA for these samples was derived from a clade 2 ER strain that according to whole genome sequence SNP analysis was closely related to the strain in the commercial erysipelas vaccine used in Sweden herein termed the “vaccine related” ER antigen. The results showed that all tested samples were clearly positive for IgY to ER and unlike the samples from flocks during and after acute erysipelas outbreaks (Fig. 2) there were no samples with titers of < 100 (Fig. 3). In most flocks, a large variation in antibody levels between individual samples was observed also in this cohort. Nonetheless, *flock* seemed to be a factor with influence on the levels of antibodies to ER with some flocks showing both higher (flocks: 20, 14, 56, 3 and 48) and lower (flocks: 29, 15 and 4) titers in comparison with each other. All flocks were considered clinically healthy but some came from farms that had experienced outbreaks of erysipelas (*n* = 11) and some of these farms also vaccinated pullets against erysipelas at placement (*n* = 6). However, no associations between levels of ER titers and either previous erysipelas outbreaks or with vaccination were identified. Likewise, no association between housing system and levels of ER titers was identified.

**Fig. 4:**
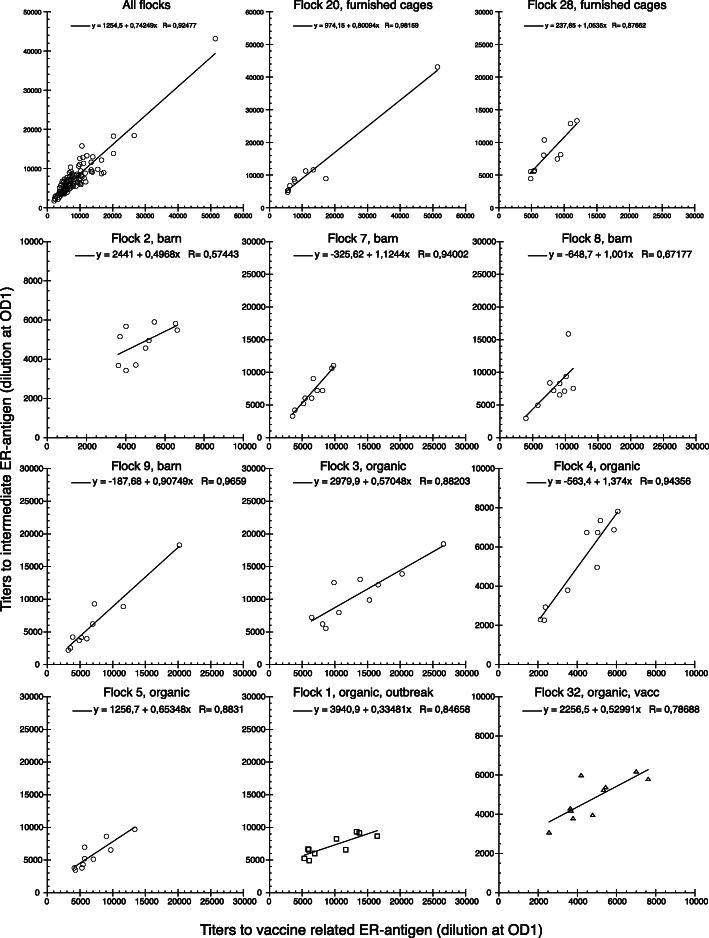
Correlation between titers to the clade 2 “vaccine related” ER antigen (x-axis) and titers to the “intermediate” ER antigen (y-axis) in individual sera for all tested samples and samples from the indicated laying hen flocks, respectively. Flock 20 and 28 were housed in furnished cages, flocks 2, 7, 8 and 9 were housed in barn production (barn) and flocks 3, 4, 5, 1 and 32 were housed in organic production with outdoor access (organic). Flocks 1 and 32 were from farms that had previous outbreaks of erysipelas (outbreak) and flock 32 was also vaccinated against erysipelas (vacc), all other tested flocks were from farms with no previous outbreaks of erysipelas. Data is shown as individual values for all tested hens (All flocks), *n* = 118, or as individual values for hens in indicated flocks with 10 ≥ n ≤ 8 for each flock. Trend lines were calculated using the curve fit linear mode in the software KaleidaGraph, version 4.1.0 (Synergy Software) and equations and R-values are shown in each panel

A subset of 118 serum samples from 11 flocks was in addition to the “vaccine related” ER antigen also tested in the ELISA using the “intermediate” ER antigen (Fig. 4). Both analysed together and grouped by flock, titers to the two ER antigens showed a positive correlation. However, for some flocks (2, 4, 1 and 32) a bias with higher titers to one of the antigens could not be excluded but the number of observations was too low for a conclusive analysis.

Thus, all hens in these healthy flocks tested at slaughter were positive for antibodies binding ER and *flock* was the only factor identified that was associated with the levels of IgY titers to ER.

## Discussion

In the present study, we evaluated the use of a modified ELISA method for detection and quantification of antibodies to ER in serum samples from laying hens in production flocks. The origin to the current ELISA method used the same protocol for ER coating antigen preparation but serum samples were only tested at one dilution, 1:100 [6, 23]. However, the relationship between the amount of target antigen and enzymatic colour reaction may only be linear in a limited interval depending on different components in the ELISA. Hence, only using one dilution may reduce the ability of the previously described protocol to quantify levels of antibodies to ER. Therefore we modified the protocol to include titration of samples to identify an interval where each sample was within the range where a linear relationship between dilution, i.e. amount of antibody, and colour reaction was achieved. This linear interval was then used to calculate a theoretical dilution that would result in an optical density of 1 using regression analysis and this dilution was defined as the titer to ER. We tested this modified ELISA protocol using serum samples from experimentally ER infected SPF-chickens [20, 21] housed under strict biosecure conditions. For these chickens we readily detected clearly increased levels of IgY to ER from approximately one week after infection which is similar to earlier reports in experimentally ER infected chickens [23]. At day 10/11, after infection we found an overall approximately 1000-fold difference between ER infected chickens and age-matched uninfected chickens and within-group differences between pre-infection levels and day 10/11 after infection of approximately 30 to 220-fold. A study using the one-dilution version of the current ELISA with antibody levels quantified as absorbance values showed an approximatly 3-fold difference in ER-antibody levels between infected and uninfected chickens at day 9 after experimental ER infection [23]. Another report using a one-dilution protocol in an ELISA system with an ER whole cell coating antigen showed an approximately 3 to 4-fold difference in ER-antibody levels between chickens vaccinated with a commercial inactivated ER-vaccine and unvaccinated chickens at 21 days after vaccination [24]. Thus, in comparison with those results, the current modified ELISA protocol noticeably improved the quantification range of ER-antibody levels in response to ER antigen exposure.

The modified ELISA protocol was then used to quantify levels of IgY to ER in serum samples from laying hens in commercial flocks either collected from flocks in association with outbreaks of acute erysipelas or collected at slaughter of flocks without health problems. This analysis showed that the majority of samples collected from erysipelas outbreaks and all of the samples collected at slaughter were positive for IgY to ER. This is in analogy with results from samples collected at slaughter of Swedish laying hens in 2005–2007 using the one-dilution version of the current ELISA to detect IgY to ER where all samples also were deemed positive [6]. In comparison, a survey of serum samples from New Zealand chickens of different age groups and housing systems, showed that 64 % of samples from chickens aged > 48 weeks were positive for antibodies to ER [19]. In addition, a study using a growth agglutination test to detect antibodies to ER showed that 5.5 % of tested laying hens reared in individual wire floor cages were positive at slaughter [18]. The difference in results from the two Swedish and the New Zealand ELISA based studies may be due to methodological differences. The New Zealand ELISA had a relatively high cut-off value, OD 1.5, based on the assumption that 16-week-old chickens from a flock with no history of erysipelas, not vaccinated against erysipelas and housed in wire floor cages were negative for ER antibodies [24]. The ELISA in the previous Swedish study had a cut-off value of OD 0.2 based on results from 7 to 9 week-old chickens from high biosecurity grandparent flocks [6] while titers from the current ELISA were judged on results from experimental ER infections of 3–4 week old SPF-chickens. However, despite differences in seroprevalence one may conclude that the accumulated serological evidence from these studies indicates a high exposure of commercial chickens to ER or other related bacteria that raise antibodies that cross-react with ER. Indeed, our current results show that chickens may respond with high titers to ER after experimental infection (i.e. in groups A-E) despite showing no clinical signs of disease and very low recovery of bacteria in blood [21]. This indicates that the ER titers observed in commercial laying hens without history of erysipelas may be due to subclinical ER infections. There are also closely related bacteria, e.g. *E. tonsillarum*, that seem to be nonpathogentic to chickens [25], and other novel *Erysipelothrix* species [26–28] with so far unknown prevalence in chickens or the chicken environment.

When comparing the current results from flocks in association with acute erysipelas outbreaks with the results from healthy flocks at slaughter, the overall mean levels of antibodies to ER were similar but in outbreak flocks samples with both very low, < 100, and very high, > 100,000, titers were observed which was not the case in the healthy flocks at slaughter. Samples with very low levels of ER antibodies were observed in flocks A1, B2, F1 and F2. Flocks B2, F1 and F2 were of a considerably younger age compared to those sampled at slaughter, which may have influenced the results. Indeed, the New Zealand survey of ER antibodies in chickens showed that seroprevalence increased significantly with increased age of the bird [19]. During the acute erysipelas outbreaks birds showing signs of disease were chosen for sampling. Hence, one may speculate that the samples with very high titers to ER reflect birds that had experienced an ongoing ER infection for long enough to mount an IgY response to it. This is also indicated by results from flock F that was sampled twice, one week apart, during the acute erysipelas outbreak where the proportion of samples with high titers was increased at the second sampling. An improved follow-up of antibody responses including measurement of ER-specific IgM in laying hens during erysipelas outbreaks is however needed to understand the kinetics of ER antibody responses in the production environment. In comparison, recurring clinical chronic erysipelas in a pig herd where pigs were positive for antibodies to ER has been reported [29].

The most striking finding from the current survey of healthy conventional laying hens was the influence of flock on the mean levels of IgY titers to ER. A large number of factors including chicken genetics, management and environment that could influence antibody levels are obviously included in ‘flock’. One explanation for varying levels of IgY titers to ER between flocks may for instance be that it reflects qualitative variations in the specificity of antibodies due to differences in the ‘flock ER profile’. A large genetic variation among ER strains has been shown [22, 30] including in genes of putative importance for immune recognition [31] and by whole genome sequencing of a large number of ER strains isolated from laying hens during outbreaks of erysipelas we have found that the genetic profile of ER is clearly associated with the farm (manuscript in preparation). Thus, the variation between flocks in levels of ER antibodies when testing with one ER strain may reflect variations in cross-reactivity of antibodies raised to different ER strains than that used to detect the antibodies. We have previously found a clear bias in IgY titers with higher titers towards the “intermediate” ER antigen compared to the “vaccine related” antigen after experimental infection of chickens with the homologous intermediate ER strain [20]. Therefore one may hypothesise that a situation with titers that are very similar to both these ER antigens, which was observed for most of flocks tested in the present study, may indicate that the antibodies were in fact raised to an altogether different ER strain or *Erysipelothrix* species or to a mixture of different strains/species.

In the present material, no association between the levels of ER titers and housing system for the conventional laying hens was found. This is in analogy with what was reported in the New Zealand survey [19] while the previous Swedish survey found significantly higher levels of antibodies to ER in sera from chickens with outdoor access compared to those housed in cage systems or in barn production (without outdoor access) [6]. Moreover, no associations between the levels of ER titers and previous outbreaks of erysipelas on the farm or with vaccination of pullets against erysipelas at placement at the farm were observed. In analogy, a report on IgY to ER in the parrot species kakapo (*Strigops habroptius*) showed that birds in their natural habitat were seropositive to ER despite no evidence of clinical disease and that antibody levels to ER increased with age of the bird [32]. Moreover, vaccination of those kakapo against ER only increased antibody levels in birds with low pre-vaccination levels of IgY to ER.

## Conclusions

The present results supports the previous observations that high numbers of commercial laying hens were seropositive to ER indicating that ER or bacteria that raises antibodies that cross-react with ER are common in this environment. The quantitative approach of the method used gives a more comprehensive picture of the antibody responses and e.g. showed a large variation in ER antibody levels between individuals where individuals with high and low titers occurred in all types of flocks. Complementary methods such as ER growth agglutination tests [18] or western blots may add more information on antibody specificity. Moreover, studies of qualitative aspects of antibodies to ER, e.g. into more detailed specificity and functional aspects such as opsonisation for phagocytosis, are needed to clarify the possible role of antibodies in protective immunity against erysipelas.

## Methods

### ELISA for detection of antibodies to ER

An earlier described [20] in-house ELISA for detection of antibodies to ER in chicken serum was used. In brief, a sonicated preparation of ER was used as coating antigen at a protein concentration of 5 µg/ml in 0.15 M Na_2_CO_3_/0.35 M NaHCO_3_ pH 9.6 coating buffer. Phosphate buffered saline (PBS) with 0.6 % bovine serum albumin (BSA; Sigma-Aldrich) was used for blocking and as diluent while PBS with 0.1 % BSA was used as a wash buffer. Horseradish peroxidase conjugated polyclonal goat anti-chicken IgG (IgY)-Fc antibodies (#AAI29P, BioRad Antibodies) were used as a tracer. Chicken sera were titrated in 2-fold steps starting at dilutions 1:100 or 1:1000 depending on antibody concentration to achieve a dilution curve. For each sample the A_450_ – A_650_ values were plotted against the sample dilution and the equation for the linear part of the curve was determined by regression analysis. Antibody titers were then calculated as the dilution that would achieve an A_450_ – A_650_ value of 1. A high titer serum sample and a negative serum sample were included on each plate as positive and negative controls for plate-to-plate variation and titers were within 1 standard deviation for inclusion.

In the present, study two ER coating antigens were used. One termed “intermediate” was prepared from ER strain 15-ALD003475 derived from an outbreak of erysipelas in a Swedish laying hen flock in 2015 as a representative of recent outbreaks in Swedish laying hens. Whole-genome SNP comparison with isolates from the study by Forde et al. [22] showed that it was of an “intermediate” lineage since it did not reveal a clear clade assignment for the strain. The “intermediate” antigen was used with samples from the experimentally infected chickens (the 15-ALD003475 strain was used for infection of these chickens), with samples from the outbreak flocks and with a subset of samples from the flocks at slaughter. The other antigen termed “vaccine related” was prepared from ER strain 13-ALD025893 that belongs to clade 2 according to the classification described by Forde et al. [22]. This strain was, according to whole genome sequence SNP analysis, closely related to ER strain M2 of serotype 2, belonging to clade 2, in the commercial vaccine used in Swedish laying flocks. The “vaccine related” antigen vas used with samples from the flocks at slaughter.

### Samples from experimentally ER infected chickens

To validate the ELISA, serum samples collected from chickens experimentally infected with ER in previously described studies [20, 21] were used. In brief, in the present study samples from a total of 102 chickens divided into six groups of 13, 18 or 19 ER infected chickens, respectively, and one group of 13 uninfected chickens were included, described in Table 1. All chickens were female Dekalb White layer hybrids purchased from a commercial hatchery (Swedfarm) and reared from day-old under SPF-conditions at the animal facilities at the National Veterinary Institute. Chickens were infected by intra-muscular injection of ER of the 15-ALD003475 strain on experimental day-0. Blood samples for serum were collected by jugular venipuncture from chickens on the experimental days indicated in Table 1. Chickens were monitored at least daily for any clinical signs of disease [20] and humane end points for severe clinical signs or prolonged moderate clinical signs were established for the experiment. All experimental chickens were killed by cervical dislocation and subjected to *post mortem* examination at the end of the experiment.

### Samples from layer flocks experiencing erysipelas outbreaks

Serum samples were collected from a selection of Swedish layer flocks during or after outbreaks of acute erysipelas during 2016 and 2017. An outbreak of erysipelas was defined as a sudden increased mortality with isolation of ER at *post mortem* examination. Blood samples were collected from hens displaying clinical signs of disease. After sample collection the hens were returned to the care of their owner. The seven different farms and nine different flocks that were included are described in Table 2.

### Samples from laying hens at slaughter

In total, 561 serum samples from the National Veterinary Institute (SVA, Uppsala, Sweden) biobank were tested. Samples were collected at slaughter of laying hens from commercial Swedish flocks without health problems. The current samples originated from a cohort of 108 flocks from which 59 flocks, 8 to 10 samples from each flock, were randomly included without records of age at sampling. For the whole cohort, age at sampling ranged from 70 to 124 weeks, with a mean of 83.0 ± 1.3 weeks (± 95 % confidence interval). Eight of the included flocks were housed in furnished cages and none of these farms had previously experienced outbreaks of erysipelas and none were vaccinated against erysipelas. Thirty-two of the included flocks were housed in barn production and three of these farms had previously experienced outbreaks of erysipelas and one flock from a previously affected farm was vaccinated with a commercial inactivated erysipelas vaccine (ER strain M2; Nobilis Erysipelas, MSD Animal Health). Nineteen of the included farms were performing organic production and flocks were housed with outdoor access and eight of these farms had previously experienced outbreaks of erysipelas and five flocks from previously affected farms were vaccinated with the commercial inactivated erysipelas vaccine.

### Data analysis

For antibody titers, geometrical mean values and 95 % confidence intervals (CI) were calculated using the software package R 3.5.0. Data were presented either as individual values, as group mean values ± CI where mean values with non-overlapping CI were treated as rejecting the null hypothesis of no difference, or as group Tukey box-plots drawn in the software KaleidaGraph, version 4.1.0 (Synergy Software). Box plots with non-overlapping maximum and minimum values were treated as rejecting the null hypothesis of no difference.

## Data Availability

All data generated and analysed in this study are included in the article text, tables and figures. The datasets used and/or analysed during the current study are available from the corresponding author upon reasonable request.

## Abbreviations

CI: Confidence intervals
ER: *Erysipelothrix rhusiopathiae*
PBS: Phosphate buffered saline
